# Five Conserved microRNAs Dominate the Small-RNA Pool of Three Arid-Zone Camel-Forage Plants: De Novo Repertoires and a Species-Matched Test of Cross-Kingdom Targeting in the Dromedary

**DOI:** 10.3390/genes17091022

**Published:** 2026-08-27

**Authors:** Maksym Zoziuk, Abdirahman Ali, Abel Dafogo Djibagao, Carla Montesano, Marina Potestà, Alessandra Minchella, Alessandro Terrinoni, Maria Cristina Caroleo, Giulia Cappelli, Dimitri Koroliouk, Mohamed Ahmed Jimale, Elena Ciani, Vittorio Colizzi

**Affiliations:** 1 Tor Vergata Foundation, 00133 Rome, Italy; dimitri.koroliouk@ukr.net (D.K.); colizzi@uniroma2.it (V.C.); 2 Department of Experimental Medicine, University of Rome Tor Vergata, 00133 Rome, Italy; montesan@uniroma2.it (C.M.); marina.pote@gmail.com (M.P.); alessandra.minchella11@gmail.com (A.M.); alessandro.terrinoni@uniroma2.it (A.T.); 3 Centre for Camel Research and Development, Faculty of Veterinary Medicine and Animal Husbandry, Somali National University KM7 Campus, Mogadishu P.O. Box 15, Somalia; abdirahman.a.ali@gmail.com (A.A.); mjimale@snu.edu.so (M.A.J.); 4 Laboratoire des Grandes Épidémies Tropicales, Faculty of Medicine, University Hospital Complex “Bon Samaritain”, N’Djamena P.O. Box 456, Chad; dafogodjibagaouabel@gmail.com; 5 Department of Health Sciences, University Magna Grecia, 88100 Catanzaro, Italy; mariacristina.caroleo@unicz.it; 6 Institute of System Biology, National Research Council, 00015 Montelibretti, Italy; giulia.cappelli@cnr.it; 7 Department of Biosciences, Biotechnologies and Environment, University of Bari, 70125 Bari, Italy; elena.ciani@uniba.it

**Keywords:** camel milk, *Camelus dromedarius*, cross-kingdom miRNA, plant microRNA, miR159, miR168a, de novo miRNA annotation, 3′-UTR length bias, milk lipogenesis, dryland food security

## Abstract

**Background**: Camel husbandry underpins food security in drylands, and dromedary (*Camelus dromedarius*) milk is valued as a functional food whose composition is thought to be shaped by the desert forage that camels browse. Dietary plant microRNAs (miRNAs) have been proposed as one molecular route linking diet to mammalian physiology, and two interactions are widely cited from experimental reports: rice miR168a repressing LDLRAP1 and plant miR159 repressing TCF7. The hypothesis remains contested, however, and it has not been tested for camel forage against camel transcripts. **Methods**: We generated de novo, hairpin-based miRNA repertoires for three arid-zone forage plants relevant to camel feeding (*Moringa oleifera*, *Ziziphus jujuba*, *Medicago sativa*) and screened the mature miRNAs against 48,746 reconstructed *C. dromedarius* 3′-UTRs under stringent thresholds, retaining one transcript per gene, weighting interactions by read abundance, normalising scores for 3′-UTR length, and testing over-representation against a matched background. The two previously reported cross-kingdom pairs served as internal positive controls and seed-level grouping as a sensitivity control. **Results**: The three forages yielded 170 hairpin-validated miRNA loci (81 *M. sativa*, 49 *Z. jujuba*, 40 *M. oleifera*) peaking at 21 nt, collapsing to 116 mature sequences and 104 seed groups. The pooled read set was strongly concentrated: five mature sequences shared by all three species carried 51% of reads, miR159 alone 31%, and 19 sequences assignable to conserved miRBase families carried 64%. The pipeline recovered the reported miR168a–LDLRAP1 pairing in the camel; the miR159–TCF7 pairing, by contrast, was not recovered, although TCF7 was among the genes targeted by other plant miRNAs. Genome-wide, predicted targeting was sparse (median 2 miRNAs per gene) and no GO, KEGG or Hallmark category was enriched at either threshold (best FDR 0.56); the nominal *p*-value distribution was approximately uniform, giving no evidence of systematic enrichment under the tested framework. Targeting multiplicity scaled with 3′-UTR length (Pearson r = 0.63; Spearman ρ = 0.58), so apparent “hub” genes are largely long-3′-UTR genes. Of 19, 12 curated milk-fat and lactation genes were among predicted targets, without over-representation (Fisher *p* = 0.36). **Conclusions**: A sensitive, species-matched analysis recovered a previously reported cross-kingdom pairing yet found no coordinated enrichment of dietary plant miRNAs on the dromedary transcriptome, and showed that an individual cross-kingdom pairing cannot be assumed to transfer between mammalian species. The work provides a first forage miRNA resource in the context of camel nutrition, sets out a reusable, species-matched analytical framework for cross-kingdom claims, and narrows future experimental work to five abundant forage-derived sequences and a short list of candidate genes (LDLRAP1, TCF7/TCF7L2, PRLR, INSR).

## 1. Introduction

Camel husbandry underpins food security across the arid belt that runs from the Sahel to Central Asia, where drought, heat, and sparse vegetation leave most other livestock unproductive [1,2]. The dromedary (*Camelus dromedarius*) tolerates water deprivation, extreme temperatures, and high dietary salinity, and it maintains milk production under conditions that strongly constrain cattle [3,4]. In pastoral systems across Chad, Somalia, and the Horn of Africa, camel milk is therefore not a niche commodity but a staple, and in prolonged dry seasons it can be an important source of animal protein, fluid, and micronutrients for herding communities [1,5].

Camel milk is also of growing interest as a functional food. Relative to bovine milk, it lacks β-lactoglobulin, has a distinct casein composition and micelle structure, a fatty-acid profile richer in long-chain unsaturated species, and elevated concentrations of lactoferrin, lysozyme, and immunoglobulins [2,3]. These properties are associated with its reported tolerability in individuals sensitive to cow-milk protein and with a range of reported metabolic effects [3,6]. Yet the substantial variation in camel-milk composition between herds, seasons, and regions is largely unexplained, and the contribution of diet to that variation has not been examined at the molecular level.

Camels are browsers rather than grazers, and the distinction matters here. Direct observation of free-ranging dromedaries in East African rangelands indicates that perennial woody plants make up close to 80% of the diet in both dry and wet seasons, and that camels spend more than four-fifths of their feeding time on dicotyledons in either season [7,8]. The diet is broad but strongly skewed: animals sample 20 to 30 species over a season, yet the 10 most preferred account for 80–87% of feeding time [8,9], so a relatively small set of plants supplies most of what enters the rumen. Daily intake is substantial, from roughly 5 to 55 kg of plant matter depending on season and availability [10]. Preference also shifts with the calendar, with Ziziphus ranking among the higher-preference browse genera in the Sahel during the dry season and Acacia species becoming prominent when rains arrive [8,9]. Whatever the season, the material a camel ingests differs fundamentally from the grass-based diet of cattle.

These observations shaped the species examined here. Ziziphus is a top-ranked browse genus for camels across African and Asian rangelands, and *Z. jujuba* is used directly as a feed in some mountainous systems [8,9]. *Moringa oleifera* is an established fodder tree in dryland agriculture and has been evaluated specifically for ruminant feeding [11]. *Medicago sativa* is a different case, and we make no claim otherwise: it is not natural browse but the principal cultivated supplement wherever camel dairying has intensified, and it was included on that basis. Species that dominate Sahelian browse in the strict sense, notably Acacia, Balanites, and Salvadora, have no healthy-leaf small-RNA data in the public archives and could not be analysed. The three species therefore represent the browse and cultivated components of a camel diet as closely as existing sequencing data allow, which is a constraint of the available data rather than a design choice.

The applied question that follows is simple: does the vegetation a camel browses leave a measurable imprint on its milk? Answering it would inform efforts to characterise, standardise, and evaluate camel-milk products, and to assess forage species as deliberate dietary interventions in dryland production.

Among the molecular routes that might connect plant diet to mammalian physiology, cross-kingdom regulation by dietary plant miRNAs is both the most discussed and the most disputed. Plant miRNAs are ~20–22-nt small RNAs, stabilised by 3′-terminal 2′-O-methylation, that direct post-transcriptional silencing through near-complementary base-pairing, predominantly within 3′-untranslated regions (3′-UTRs) [12,13,14]. Because their pairing is extensive rather than seed-restricted [15,16], a plant miRNA that encounters a sufficiently complementary mammalian 3′-UTR could in principle repress that transcript. The idea is therefore worth testing rather than dismissing.

Several influential studies have reported such effects. Exogenous rice miR168a was described as surviving digestion, entering the circulation and repressing hepatic LDLRAP1 [17]; honeysuckle miR2911 was reported to suppress influenza-A replication in mice [18]; and plant miR159 was detected in human serum, its abundance inversely correlated with breast-cancer progression, with a synthetic mimic reported to inhibit xenograft tumour growth through the Wnt transcription factor TCF7 [19]. Conserved plant miRNA families, among them miR166, miR156, miR157, miR172 and miR168, have also been detected in human and livestock milk and in milk-derived extracellular vesicles [20,21,22,23,24,25,26], which provides a plausible vehicle for transfer between mother and offspring and, by extension, between forage and consumer.

An equally substantial literature disputes these findings. Controlled feeding experiments have failed to detect meaningful intestinal uptake of plant miRNAs [27,28,29]; systematic re-analyses have attributed many reported dietary plant-miRNA signals to index hopping, cross-mapping or environmental contamination [30,31,32,33]; and quantitative arguments hold that the copy numbers reaching mammalian tissues fall well below the stoichiometry required for repression [21,34]. Cross-kingdom miRNA regulation is therefore best treated as an open question with credible evidence on both sides, and in that situation a well-controlled test is worth more than another positive report.

Two recurrent weaknesses have, however, biased computational assessments toward positive conclusions. First, plant miRNAs are commonly mapped by homology to human miRBase entries and screened against human predicted targets, so the mammalian species of interest is absent from the analysis and species-specific 3′-UTR variation is discarded. Second, permissive alignment thresholds applied to large target lists generate apparent convergence that is difficult to separate from the null expectation, because predicted-target counts scale with 3′-UTR length [35,36,37]: genes with long 3′-UTRs accumulate predicted regulators irrespective of biology. To our knowledge, these two confounds have not been jointly controlled in a livestock species, and this is the gap the present design was built to address.

**Figure 1:**
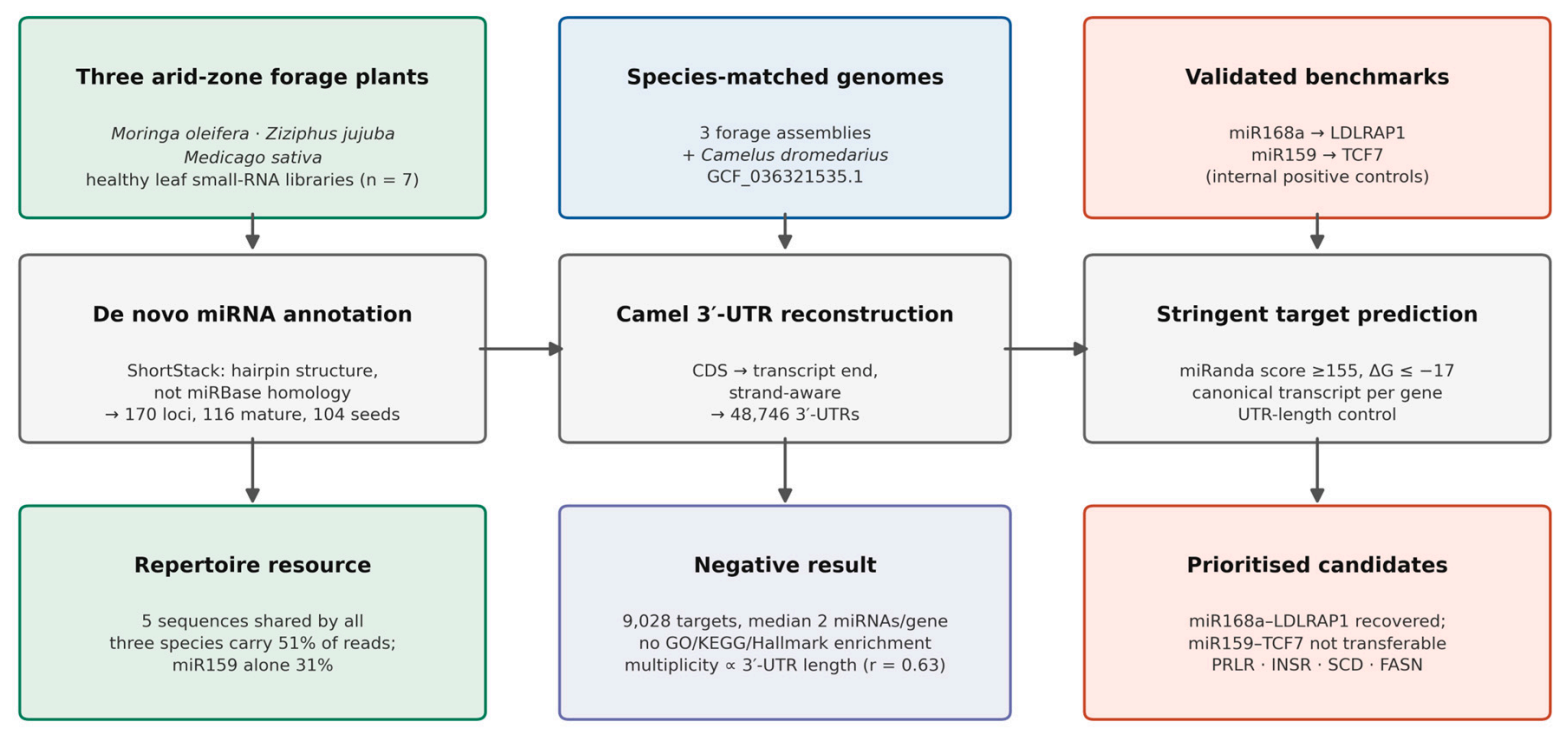
Study design and analytical logic. Three arid-zone forage plants relevant to dromedary feeding were selected on the joint criteria of documented consumption and availability of healthy-leaf small-RNA data; miRNAs were annotated de novo from hairpin structure rather than by homology, so that species-specific sequences absent from miRBase could be recovered. Targets were predicted against reconstructed 3′-UTRs of the dromedary genome rather than human surrogates, under stringent thresholds, with one transcript per gene and per-kilobase length normalisation of Global Scores. The two previously reported mammalian cross-kingdom interactions (miR168a–LDLRAP1, miR159–TCF7) were used as internal positive controls, so that a negative genome-wide result could be distinguished from insufficient sensitivity. The three intended outputs are a reusable forage miRNA resource, a controlled negative result with a candidate mechanistic explanation, and a prioritised candidate list for experimental work.

The study was organised around four deliberate choices intended to remove these confounds (Figure 1). We selected forage species on the joint criteria of documented camel consumption and availability of healthy-leaf small-RNA data, since stressed or diseased tissue distorts miRNA profiles and libraries that differ in pathology cannot be compared cleanly. We annotated miRNAs de novo from hairpin structure rather than by homology [15,38,39], because the forage species are absent from miRBase and homology-based selection would recover only sequences already known from other plants. We predicted targets against reconstructed *C. dromedarius* 3′-UTRs rather than human surrogates, so that any inference applies to the animal of interest. Finally, we used the two previously reported cross-kingdom interactions as internal positive controls, so that a negative genome-wide result could be distinguished from simple insensitivity. The aim is deliberately conservative: to establish what cross-kingdom signal survives careful analysis, and to delimit computational inference before costly experimental work on camel milk is undertaken.

## 2. Materials and Methods

### 2.1. Forage-Plant Selection and Datasets

Species were chosen on two joint criteria. The first was documented consumption by dromedary camels, since the question concerns the animal’s actual diet rather than plants of general pharmacological interest [7,11]. The second was availability of raw small-RNA sequencing from healthy, unstressed leaf tissue, because miRNA profiles respond strongly to biotic and abiotic stress [16]: libraries from callus, cold-treated or phytoplasma-infected material describe a perturbed plant, not the forage a camel eats. Applying both criteria yielded *Moringa oleifera*, *Ziziphus jujuba*, and *Medicago sativa* (alfalfa). Species prominent in Sahelian camel diets, such as Acacia spp., Balanites aegyptiaca, and Salvadora persica, were considered but could not be included because no healthy-leaf small-RNA data exist for them; this is a property of the public data, not of the design, and is revisited in Section 4.7.

Libraries were retrieved from the NCBI Sequence Read Archive and, in each case, the sample annotated as healthy leaf in the source study was used. For *M. oleifera*, three leaf libraries were taken from a study that also profiled callus (SRR7770291–SRR7770293; PRJNA488608 [40]); only the leaf libraries were used. For *Z. jujuba*, the healthy wild-type leaf library was taken from a study of witches’-broom phytoplasma response (SRR4334534; SRP090598 [41]); the infected samples from that study were excluded. For *M. sativa*, three leaf libraries across two cultivars were used (SRR1201405–SRR1201407; SRP040470). One alfalfa archive (SRR1201408) failed integrity checks on repeated download and was discarded rather than partially recovered, since a truncated archive would bias abundance estimates in an unquantifiable direction. Accession, tissue, cultivar, and treatment for every library are listed in Table 1 and Supplementary Table S1. The three species differ in replication (three leaf libraries for *M. oleifera* and *M. sativa*, one for *Z. jujuba*) and in cultivar, and abundance estimates are therefore reported at the pooled level and interpreted with this imbalance in mind (Section 4.7).

**Table 1:** Small-RNA datasets and read-processing quality control. “Passed reads” are 18–26 nt reads retained after adapter trimming and quality filtering; “Peak (nt)” is the modal trimmed-read length; “miRNA (%)” is the fraction of passed reads mapping to a miRBase mature sequence. Full metrics, including with-adapter and length-filtered counts, are in Supplementary Table S1.

Run	Species	Sample/Cultivar	Raw Reads	Passed Reads	Passed (%)	Peak (nt)	miRNA (%)
SRR7770291	*M. oleifera*	leaf rep1	24,996,624	5,729,028	22.9	21	2.6
SRR7770292	*M. oleifera*	leaf rep2	22,743,760	4,135,123	18.2	21	2.2
SRR7770293	*M. oleifera*	leaf rep3	24,160,017	7,565,465	31.3	19 *	10.3
SRR4334534	*Z. jujuba*	healthy WT leaf	14,171,805	7,812,588	55.1	21	7.1
SRR1201405	*M. sativa*	leaf, cv. DS	7,035,161	3,369,235	47.9	21	2.0
SRR1201406	*M. sativa*	leaf, cv. NM	6,765,502	3,336,696	49.3	24 *	2.9
SRR1201407	*M. sativa*	leaf, cv. NS	7,145,001	2,402,795	33.6	24 *	2.3

**Figure 2:**
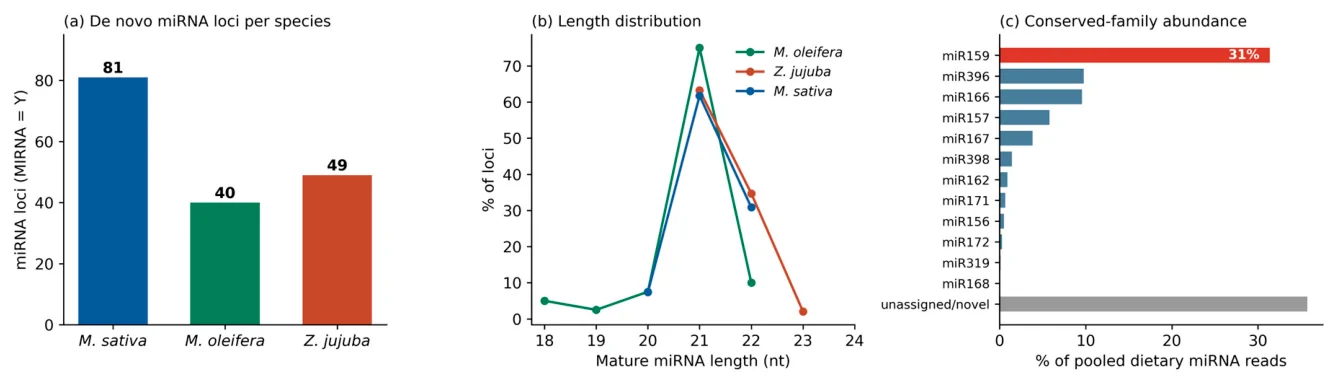
De novo microRNA repertoires of three arid-zone camel-forage plants. (**a**) Number of hairpin-validated miRNA loci per species (ShortStack, MIRNA = Y); 170 loci in total. (**b**) Length distribution of mature sequences at the miRNA loci; all three species peak sharply at 21 nt, consistent with the canonical plant-miRNA size. (**c**) Relative abundance of conserved miRNA families in the pooled forage read set, as the summed percentage of pooled reads over the mature variants assigned to each family. The distribution is highly unequal: miR159 alone (red) contributes 31% of reads, and sequences assignable to conserved families contribute 64%. The dominant families are those previously detected in mammalian milk and serum.

* The modal trimmed-read length reflects the whole small-RNA population and is dominated in some libraries by non-miRNA species; the miRNA loci themselves peak at 21 nt in every species (Figure 2b).

### 2.2. Read Processing and Quality Control

Adapters were identified per library from the most over-represented 3′-terminal k-mer (k = 12) among the first 200,000 reads, rather than assumed from the reported protocol, and removed with Cutadapt v4.4 [42] (options -q 20, --minimum-length 18, --maximum-length 26, --discard-untrimmed). Reads of 18–26 nt passing a Phred quality cut-off of 20 were retained; untrimmed reads were discarded. Per-library adapter sequences, retention rates, and modal trimmed-read lengths are given in Table 1 and Supplementary Table S1, and the modal length serves as an independent check that adapter assignment was correct. Reads outside the 20–24 nt Dicer window were retained at this stage but were not called as miRNA loci by the structural annotation described in Section 2.3.

### 2.3. De Novo miRNA Identification

Homology-based annotation was rejected for a specific reason: none of the three forage species has miRNAs deposited in miRBase, so homology would recover only sequences already described in other plants, would classify near-variants of a family as separate entities or discard them, and would systematically miss species-specific sequences. Mature miRNAs were therefore identified de novo from hairpin secondary structure with ShortStack v4.1.2 [38,39], which applies the community criteria for plant miRNA annotation [15] and, in doing so, excludes the abundant 24-nt heterochromatic siRNA fraction that dominates plant small-RNA libraries. Trimmed reads of each species were aligned with Bowtie v1.3.1 [43] to its own reference genome (*M. oleifera*, GCA_021397835.1; *Z. jujuba*, GCF_000826755.1 [44]; *M. sativa*, GCF_051527215.1). Clusters were called across 20–24 nt Dicer sizes at a minimum coverage of two reads, with secondary structure evaluated by RNAfold (ViennaRNA v2.5.1) [45]. Only loci flagged MIRNA = Y were retained; this structural flag, not read depth, is the basis for retention, and the loci are accordingly described as hairpin-validated rather than high-confidence. Homology to miRBase v22 [46] was used only to assign conserved family names post hoc, by exact match of the mature sequence to a miRBase family, and never to select or filter loci. Identical mature sequences arising at separate genomic loci were collapsed with summed read support, since they represent one regulatory sequence; the resulting count of unique mature sequences is therefore smaller than the number of genomic loci.

### 2.4. Seed-Level Sensitivity Control

Because near-identical isomiRs sharing a 5′ seed could inflate apparent repertoire diversity and, in principle, split the read support of a single regulatory activity across several entries, mature sequences were additionally grouped by seed (positions 2–8) with summed abundance, and the target and enrichment analyses were repeated at seed-group level. This grouping is a sensitivity check rather than a biological family assignment: because plant miRNA pairing is not seed-restricted, a seed group can combine sequences with different targeting behaviour, and the two levels of analysis were compared for target counts, the 3′-UTR-length relationship, and enrichment outcomes (Section 3).

### 2.5. Camel 3′-UTR Reconstruction

The annotated *C. dromedarius* assembly GCF_036321535.1 (mCamDro1.pat) was used [47]. Because this annotation contains no explicit three_prime_UTR features, a common situation for non-model livestock genomes, 3′-UTRs were reconstructed per transcript as the strand-aware mRNA interval downstream of the annotated CDS terminus, yielding 48,746 sequences; coordinates were parsed with gffread (v0.12.7) [48], intervals extracted with bedtools getfasta (v2.30.0) [49], and the genome indexed with SAMtools (v1.17) [50]. For each gene, the transcript with the longest reconstructed 3′-UTR was retained, to give one representative interval per gene. Reconstructed 3′-UTRs exceeding 10 kb (362 genes) were removed as annotation artefacts (Section 3.3), leaving 17,876 genes with a usable 3′-UTR. The 3′-UTR was chosen as the search space because plant and animal miRNAs act predominantly there [12,51]; coding-region and 5′-UTR sites were not assessed, a scope limitation revisited in Section 4.7. Selecting the longest available interval maximises the search space per gene and is the more conservative choice for a study whose central result is negative, but it also inherits transcript-end error and is discussed as such in Section 4.7.

### 2.6. Cross-Kingdom Target Prediction

Targets were predicted with miRanda v3.3a [35,36], chosen because its alignment model scores complementarity along the full length of the pairing rather than from a fixed seed, which matches the extensive, non-seed-restricted pairing mode of plant miRNAs; seed-only mammalian predictors calibrated on conserved 7-mer sites [37] are not appropriate for exogenous plant sequences. Thresholds were set stringently (alignment score ≥ 155, minimum free energy ≤ −17 kcal/mol, with the miRanda strict-seed option enabled) on the reasoning that a biologically plausible cross-kingdom site in a mammal should resemble a strong, near-complementary site. Requiring 5′-seed complementarity in addition to the score and energy cut-offs is a conservative filter: it can exclude genuinely plant-type, non-seed sites and therefore biases the analysis toward fewer predicted targets, which makes a negative genome-wide result conservative rather than inflated; this trade-off is noted as a source of false negatives in Section 4.7. Permissive settings (score ≥ 140, energy ≤ −10 kcal/mol, all other parameters identical) were run in parallel and are reported for comparison. Two further filters were applied: one representative transcript per gene (Section 2.5), and, per miRNA–gene pair, only the best-scoring site; the resulting multiplicity therefore counts distinct miRNAs per gene, not distinct sites. The exact command lines and software versions are deposited with the analysis code (Data Availability).

Screening the 116 mature miRNAs against the reconstructed 3′-UTRs predicted at least one interaction for 9028 genes, of which 8152 (90%) carried an orthologous gene symbol usable for functional analysis; the remaining 10% carried only locus identifiers and were excluded from the enrichment tests.

### 2.7. Abundance-Weighted Scoring

Two metrics were kept distinct. Targeting multiplicity—the number of distinct plant miRNAs predicted to bind a gene—is the primary convergence metric and defines the gene sets used in the enrichment tests (Section 2.8). A Global Score is reported separately as a secondary prioritisation metric for ranking candidate genes. For each targeted gene g, the Global Score was W_x_ = Σ_i_ E_i_ · (S_i_/S_max_)^0.7, where E_i_ is the relative read abundance (%) of miRNA i in the pooled forage read set, S_i_ is the miRanda alignment score of the miRNA i–gene g site, and S_max_ is the maximum miRanda alignment score observed across all retained miRNA–gene pairs. The exponent 0.7 is a heuristic weighting that compresses high-scoring outliers so that a single strong site does not dominate a gene’s score; it is not a fitted parameter. Read abundance is weighted because a highly represented forage miRNA is a more plausible effector than a rare one, with the caveat that read abundance in pooled leaf libraries is not a measurement of dietary exposure. Global Scores were normalised per kilobase of 3′-UTR, because predicted-target counts scale with 3′-UTR length [35,36,37] and unnormalised ranking would simply recover the longest transcripts.

### 2.8. Functional Analysis, Controls, and Statistics

Over-representation of Gene Ontology Biological Process [52], KEGG [53] and MSigDB Hallmark [54] terms was tested with Enrichr through GSEApy v1.1.3 [55,56], using the GO_Biological_Process_2021, KEGG_2021_Human, and MSigDB_Hallmark_2020 libraries (accessed March 2025). The background was the complete set of targeted, symbol-bearing genes (*n* = 8152) rather than the whole genome, because a genome background would test whether targeted genes differ from all genes—a comparison that 3′-UTR length would confound—rather than whether multiply-targeted genes differ from targeted genes in general. Two targeting thresholds (≥5 and ≥10 miRNAs per gene) were examined to avoid dependence on a single cut-off, and significance was assessed at Benjamini–Hochberg FDR < 0.05 [57]. Because a null FDR result can arise either from absence of signal or from insufficient power, the distribution of nominal *p*-values was additionally inspected; an approximately uniform distribution is the expected behaviour under the null, whereas a genuine but weak enrichment would leave an excess of small *p*-values. This diagnostic was applied to the GO Biological Process results, the largest term set. A curated set of 19 milk-fat-synthesis and lactation genes, defined from a bovine reference network [58] and used here as a cross-species functional proxy, was tested by two-sided Fisher’s exact test against all camel genes with a reconstructed 3′-UTR (*n* = 17,876). The two genes with previously reported plant-miRNA regulation in mammals, LDLRAP1 [17] and TCF7 [19], were examined as internal positive controls, both at the gene level and for the specific cognate miRNA. The relationship between targeting multiplicity and 3′-UTR length was quantified by Pearson correlation and, because multiplicity is a count and 3′-UTR length is right-skewed, by Spearman’s rank correlation as a non-parametric check. Analyses used Python 3.10; code, parameters, software versions, and intermediate tables are deposited at https://github.com/maksimkamekspeks/camel-forage-mirna-crosskingdom (accessed on 24 August 2026), 10.5281/zenodo.21938711.

### 2.9. Robustness: Threshold Sensitivity and a Matched Null Model

Two additional analyses were run to test whether the genome-wide picture depends on the chosen thresholds or on target-list size. First, targeting was recomputed across a grid of miRanda thresholds (alignment score 140–165, minimum free energy −10 to −20 kcal/mol), and at each grid point the number of targeted transcripts, the median, and maximum targeting multiplicity, and the Pearson correlation between multiplicity and 3′-UTR length were recorded (Supplementary Table S6). Second, to test whether the observed multiplicity concentration and cross-species target overlap exceed what target-list size alone would produce, each miRNA was assigned a random target set of its observed size under two null models: a uniform model (transcripts drawn with equal probability) and a length-matched model (transcripts drawn with probability proportional to 3′-UTR length), each repeated 200 times with a fixed random seed (Supplementary Table S7; Figure S1). This is the computable form of a target-list-size control; a complementary null that shuffles the miRNA sequences themselves and re-runs miRanda is provided in the deposited pipeline. Both analyses were performed at transcript level, before the per-gene longest-3′-UTR collapse, so that each interval contributes its own length.

## 3. Results

### 3.1. Five Sequences Carry Half of the Pooled Forage Read Set

Structure-based annotation recovered 170 hairpin-validated miRNA loci: 81 in *Medicago sativa*, 49 in *Ziziphus jujuba*, and 40 in *Moringa oleifera* (Figure 2a; Supplementary Table S2). To our knowledge, this is the first analysis of these plant miRNA repertoires in the context of camel forage. Mature lengths peaked sharply at 21 nt in every species (Figure 2b), consistent with the canonical plant-miRNA size and with structural capture of miRNA loci rather than degradation fragments or the 24-nt heterochromatic siRNA fraction. Collapsing identical mature sequences that arose at different genomic loci gave 116 unique mature sequences, which resolved into 104 seed groups; only 11 groups contained more than one variant, so the repertoire is not an artefact of isomiR splitting, and the target and enrichment analyses gave the same outcome at seed-group level (Section 3.2 and Section 3.3).

The most consequential feature of the repertoire is its inequality (Figure 2c; Table 2). Of the 116 unique sequences, 19 could be assigned by exact match to a conserved miRBase family, and these 16% of sequences carried 64% of all reads. More strikingly, five mature sequences present in all three species together accounted for 51% of the pooled forage read set: miR159 (31%), the dominant miR166 variant (9.4%), two miR396 variants (9.8% combined), and a miR167 variant (0.7%). Because the three plants belong to three distantly related families (Moringaceae, Rhamnaceae, and Fabaceae), this shared dominance reflects the deep conservation of plant miRNAs rather than shared ancestry of the forages, and it means the three species contribute a common set of abundant sequences to whatever a camel browses. We emphasise that read abundance in pooled leaf libraries is not a measurement of how much of each sequence a camel ingests, which depends on intake, digestion, and survival through the gut.

This has a direct bearing on the cross-kingdom question. The abundant conserved families identified here are the same families repeatedly reported in mammalian milk, serum, and milk-derived extracellular vesicles [20,21,22,23,24,25,26], and miR159 is among the best-studied plant miRNAs reported to act in mammalian systems [19]. An experimental test of transfer therefore need not survey hundreds of sequences: assays for approximately five sequences would cover half of the pooled forage reads. Alongside this conserved core, a single sequence not assignable to any miRBase family accounted for 15.5% of the pooled reads (29% of the *M. oleifera* reads); it is reported as an unassigned candidate rather than a validated novel miRNA, and its high representation from a single library warrants direct scrutiny before it is treated as a genuine species-specific miRNA.

### 3.2. Positive Controls: One Reported Interaction Recovered, One Not

Screening the 116 mature miRNAs against the reconstructed camel 3′-UTRs under stringent thresholds predicted interactions for 9028 camel genes, 8152 of which carried an orthologous symbol (Supplementary Table S3). Before interpreting the genome-wide picture, we asked whether the analysis could recover cross-kingdom interactions that have been reported experimentally in mammals, since a negative result from an insensitive method would be uninformative.

The two controls behaved differently (Table 3). The rice miR168a–LDLRAP1 interaction [17] was recovered: a mature sequence identical to miR168 was present in the *M. sativa* repertoire and was among five plant miRNAs predicted to bind the camel LDLRAP1 3′-UTR. Recovering a previously reported cross-kingdom pairing in a non-model species, under stringent thresholds and without homology-based assistance, shows that the analysis can detect a genuine near-complementary site at the sequence level; it does not, on its own, establish that the interaction is functional in the camel. The miR159–TCF7 interaction [19], by contrast, was not recovered as a specific pair: although TCF7 was among the more heavily targeted camel genes (14 plant miRNAs predicted) and miR159 was the most abundant forage sequence, miR159 itself was not predicted to bind the selected camel TCF7 3′-UTR.

This asymmetry is itself informative. It indicates that an individual cross-kingdom pairing demonstrated in one mammal cannot be assumed to operate in another, because 3′-UTR sequences diverge between species even where the coding sequence is conserved and the gene is functionally equivalent. Non-recovery here has several possible causes—sequence divergence at the site, the particular reconstructed transcript, or the stringent thresholds—and the sequence-level comparison needed to distinguish them is the natural next step (Section 4.7). What the result already shows is that analyses mapping plant miRNAs onto human targets and extrapolating to livestock risk reporting pairings that do not exist in the animal of interest are a confound that cannot be seen at all without a species-matched target set.

### 3.3. Sparse Targeting, No Detectable Enrichment, and a Length Relationship

**Figure 3:**
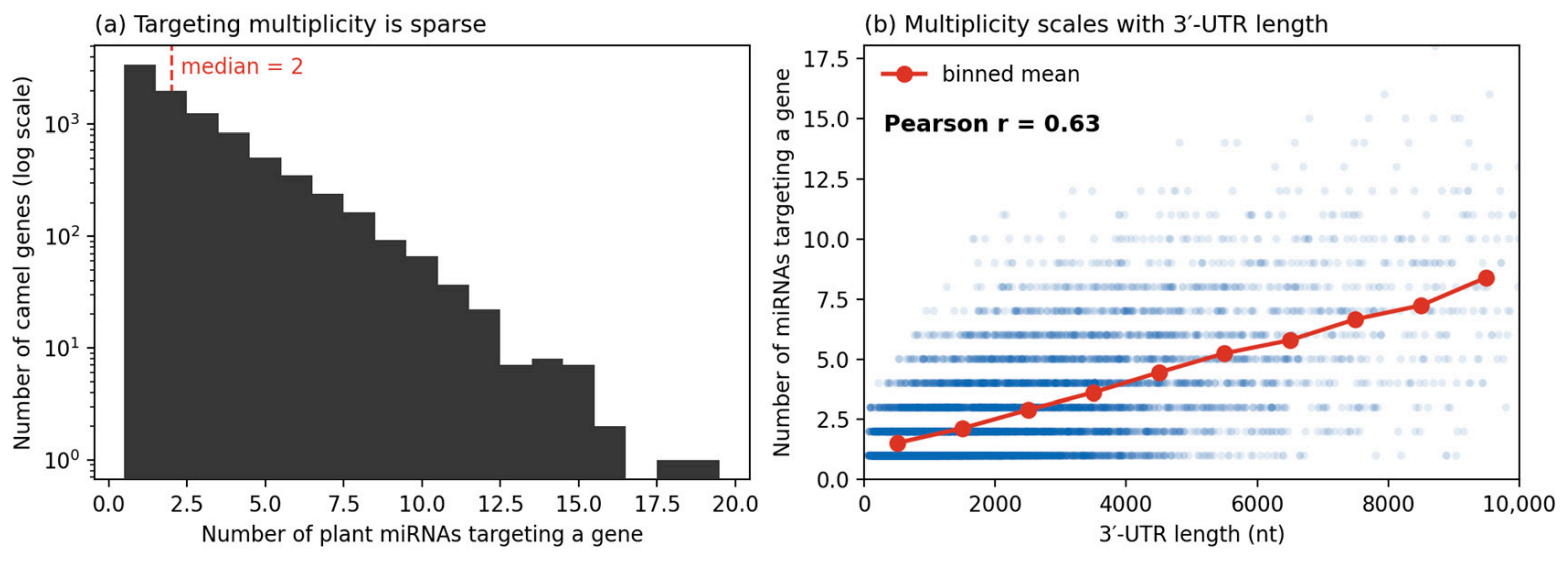
Predicted targeting multiplicity increases with 3′-UTR length. (**a**) Distribution of targeting multiplicity: number of camel genes (log scale) bound by a given number of plant miRNAs under stringent thresholds. Targeting is sparse, with a median of two miRNAs per gene and no gene bound by 20 or more. (**b**) Targeting multiplicity against 3′-UTR length for all 9028 targeted genes, with binned means in red. Multiplicity rises with 3′-UTR length (Pearson r = 0.63; Spearman ρ = 0.58), so apparent miRNA “hub” genes are largely long-3′-UTR genes. This relationship is consistent with length-driven target accumulation and is the reason Global Scores were normalised per kilobase.

Genome-wide, predicted targeting was sparse rather than saturating: the median camel gene was bound by 2 plant miRNAs, 1511 genes by 5 or more, 151 by 10 or more, and none by 20 or more (Figure 3a). Permissive thresholds, by contrast, produced 187,966 miRNA–gene pairs, with individual genes apparently bound by up to 98 of the 116 miRNAs—a density implausible enough to motivate the stringent analysis.

**Figure 4:**
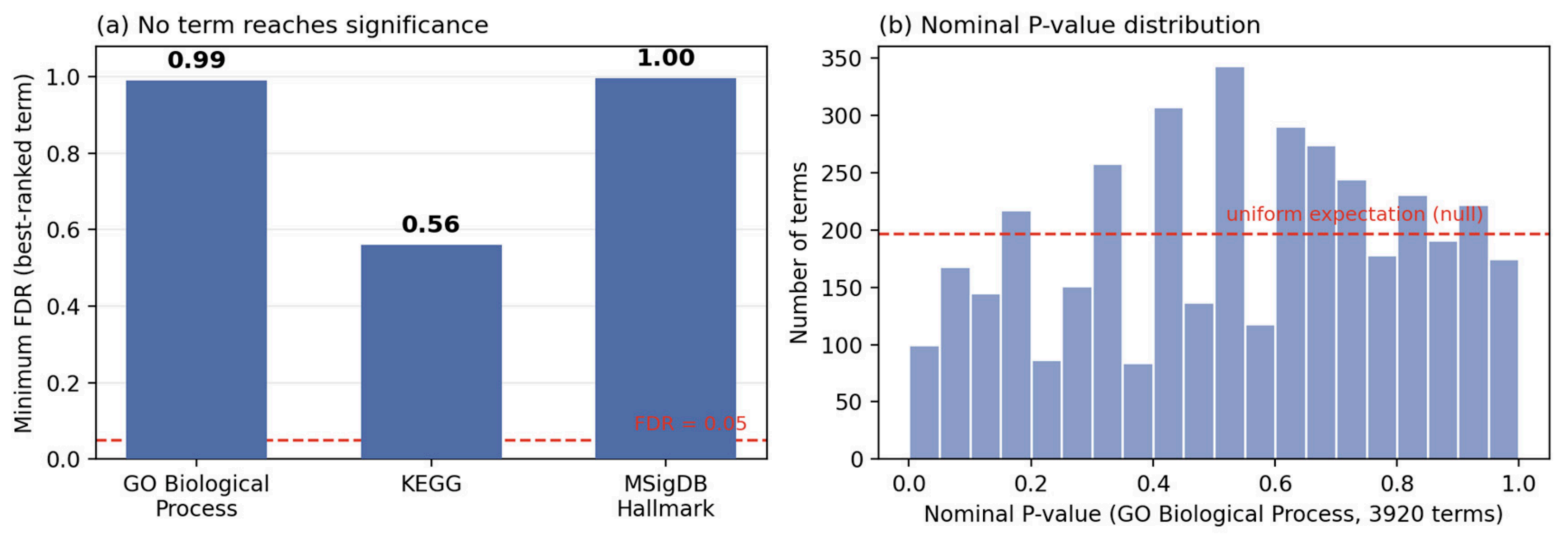
No significant enrichment was detected across the tested gene-set libraries. (**a**) Minimum Benjamini–Hochberg FDR attained by the best-ranked term in each library, for multiply-targeted genes tested against the background of all targeted genes; no term approaches the significance threshold (red line, FDR = 0.05). (**b**) Distribution of nominal *p*-values across the GO Biological Process terms. The distribution is approximately uniform, matching the null expectation (red line); a genuine but weak enrichment would produce an excess of low *p*-values. The absence of such an excess indicates that multiply-targeted genes are not concentrated in the tested pathways beyond chance.

Over-representation analysis against the background of all targeted genes returned no significant term in any library at either threshold, with minimum Benjamini–Hochberg FDR values of 0.99 (GO Biological Process), 0.56 (KEGG) and 1.00 (Hallmark) (Figure 4a; Supplementary Table S5). The distribution of nominal *p*-values across the GO Biological Process terms was approximately uniform (Figure 4b), the expected behaviour under the null hypothesis; a genuine but weak enrichment would instead leave a visible excess of small *p*-values even where FDR correction removed individual significance. Taken together, these results give no evidence of systematic enrichment under the tested framework, rather than an enrichment that the test was merely underpowered to detect. We nonetheless note that this diagnostic speaks to conserved, human-annotated gene sets; camel-specific functions that are poorly represented in those libraries would not be captured (Section 4.7).

The data also indicate why permissive analyses report convergence. Even under stringent thresholds, the number of miRNAs predicted to target a gene remained correlated with the length of its 3′-UTR (Pearson r = 0.63; Spearman ρ = 0.58; Figure 3b): genes that appear to be miRNA “hubs” are predominantly genes with long 3′-UTRs, which offer more sequence over which chance complementarity can occur [36,37,51,59,60]. Under permissive settings, the effect was extreme, with the most heavily targeted genes carrying reconstructed 3′-UTRs of 100–500 kb—far beyond the range expected for annotated mammalian 3′-UTRs, and strongly suggestive of transcript-end annotation artefacts; the 362 genes with reconstructed 3′-UTRs above 10 kb were excluded on this basis. The apparent regulatory programmes reported by permissive, human-surrogate analyses can therefore arise from sequence opportunity associated with 3′-UTR length, without any underlying biological signal.

### 3.4. A Prioritised Candidate List for Camel-Milk Biology

**Figure 5:**
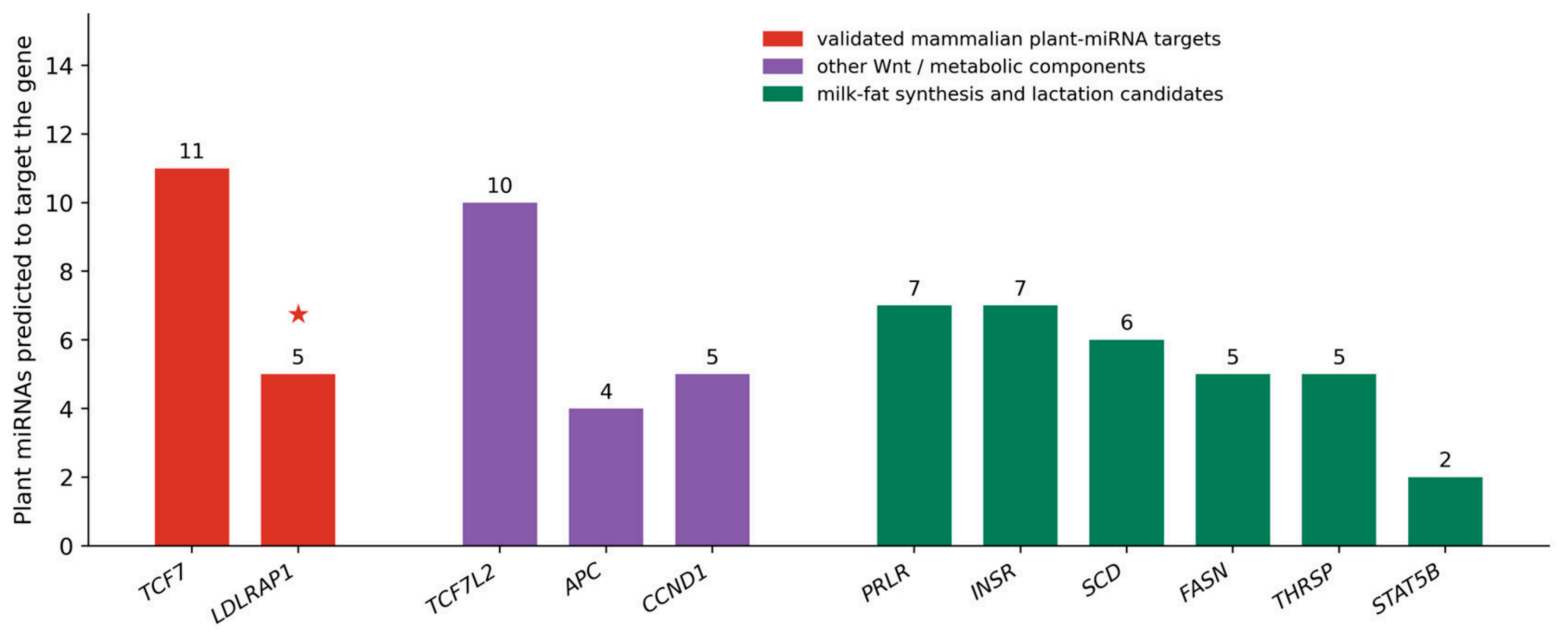
Predicted targets include previously reported cross-kingdom loci and prioritised candidates for milk biology. Bars show the number of plant miRNAs predicted to bind each camel gene under stringent thresholds; these are predicted sequence interactions, not experimentally validated regulation. Red: the two mammalian genes with previously reported plant-miRNA regulation; the star marks LDLRAP1, whose reported partner miR168a was recovered in the camel; TCF7 was targeted by 14 other plant miRNAs but not by miR159 itself. Purple: additional Wnt and metabolic components. Green: curated milk-fat-synthesis and lactation genes, headed by the prolactin and insulin receptors. The lipogenic set as a whole was not statistically over-represented (Fisher’s exact *p* = 0.36).

No category was statistically enriched, so the genes below are reported strictly as hypothesis-generating candidates, not as an enriched pathway. They were selected on two explicit criteria: relevance to lactation or milk-lipid biology as defined in advance from a bovine reference network [58], and either recovery of a previously reported cross-kingdom locus or above-median targeting multiplicity. On this basis, two groups warrant attention for the applied question. The reported cross-kingdom locus TCF7 and its paralogue TCF7L2 (10 miRNAs)—a Wnt effector implicated in insulin secretion and, in human genetics, in type-2-diabetes risk—were both among the more heavily targeted genes, together with further Wnt and metabolic components including APC, CCND1 and GSK3B (Figure 5). Separately, 12 of 19 curated milk-fat-synthesis and lactation genes were among predicted targets (Table 4), headed by the prolactin receptor (PRLR, 7 miRNAs) and insulin receptor (INSR, 7), key endocrine receptors in lactogenic and metabolic signalling, followed by the lipogenic enzymes SCD (6) and FASN (5), the lipogenic regulator THRSP (5), and ACACA, ACACB, LPL, GPAM, STAT5B and the milk-fat-globule genes XDH, and BTN1A1 [58].

This curated set was not over-represented relative to the genome-wide targeting rate: 12 of 19 genes were targeted (63.2%) against 50.5% of all camel genes with a reconstructed 3′-UTR, giving a two-sided Fisher’s exact odds ratio of 1.68 (*p* = 0.36). We therefore make no claim of pathway enrichment, and the apparent coherence of a “lipogenic module” among targets should not be read as coordinated regulation. The value of the list is operational: it converts a diffuse genome-wide question into a small number of specific, mechanistically interpretable diet–gene links that can be assayed directly in mammary tissue and milk, subject to confirmation that the target transcripts are expressed there.

### 3.5. The Negative Picture Is Stable Across Thresholds and Matched to a Length Null

Two robustness checks support the interpretation above. Across the full grid of miRanda thresholds (score 140–165, energy −10 to −20 kcal/mol), the median transcript remained bound by only one to four plant miRNAs, and the correlation between targeting multiplicity and 3′-UTR length stayed strong at every setting (Pearson r = 0.75–0.94; Supplementary Table S6; Figure S1a). No threshold produced a qualitatively different, saturating or biologically structured picture; tightening the thresholds simply scaled the whole target set down. The sparse targeting and the length relationship are therefore properties of the data rather than artefacts of a particular cut-off.

The matched null model addresses whether the apparent convergence exceeds target-list size (Supplementary Table S7; Figure S1b). At the stringent threshold, transcripts targeted by 10 or more plant miRNAs numbered 1415, observed, against roughly 5 expected under a uniform size-matched null but about 1025 under a null that additionally matches 3′-UTR length—so most of the multiplicity concentration is a length effect, not random chance and not organised biology. The same holds for cross-species overlap: 8403 transcripts were predicted targets of at least one abundant miRNA from each of the three species, against about 8031 ± 56 expected from the length-matched null, meaning that close to all of the cross-species overlap is explained by target-list size and 3′-UTR length once these are controlled. A small residual above the length-matched null is consistent with the abundance skew toward the shared conserved families, but it is not accompanied by any gene-set enrichment (Section 3.3). Together with the threshold sweep, this indicates that the convergence reported by permissive, human-surrogate analyses can be reproduced by sequence opportunity alone.

## 4. Discussion

### 4.1. A Concentrated Read Set Makes the Hypothesis Experimentally Tractable

The most immediately useful outcome of this work is not the negative result but the structure of the forage miRNA pool. Five sequences shared by three distantly related plant families account for half of all pooled forage reads, and nineteen conserved sequences for nearly two-thirds. Cross-kingdom transfer has been difficult to test partly because the candidate space seemed unbounded; these data show that it is not. The three forages contribute a common set of abundant sequences, headed by miR159, among the best-studied plant miRNAs reported to act in mammals [19], and by miR166 and miR396, families repeatedly detected in mammalian milk and serum [20,21,22,23,24,25,26]. An experimental test of whether dietary plant miRNAs reach camel tissues therefore requires assays for roughly five sequences rather than a survey of hundreds, which brings the question within reach of a modest targeted study. Read abundance is not itself a measure of dietary exposure, so these five are a starting point for measurement, not a claim about ingested quantities.

### 4.2. Species-Matched Analysis Changes the Answer

The internal positive controls give the study’s most transferable methodological finding. Recovery of the reported miR168a–LDLRAP1 interaction [17] in the camel, from de novo annotated forage miRNAs and under stringent thresholds, shows that the analysis recovers a genuine near-complementary site rather than suppressing all signal through conservative thresholds; it is a sequence-level control, not evidence that the interaction is functional in the camel. Non-recovery of miR159–TCF7 [19] is equally informative: the cognate miRNA was the most abundant sequence in the forage and the target gene was among the more heavily targeted in the camel, yet the specific pairing was not predicted for the selected camel transcript. The most parsimonious explanation is divergence of the 3′-UTR between mammalian species even where coding sequence and gene function are conserved, though the reconstructed transcript and the stringent thresholds are alternatives that a direct sequence comparison would separate (Section 4.7). The practical implication is that cross-kingdom interactions reported in humans or rodents cannot be assumed to operate in livestock, and predictions made against human 3′-UTRs may describe pairings that do not exist in the target species.

### 4.3. Why the Negative Result Is Consistent Across the Tested Analyses

Against this internal benchmark of sensitivity, the absence of detectable enrichment is meaningful within its scope. No GO, KEGG, or Hallmark category was enriched at either targeting threshold, and the approximately uniform distribution of nominal *p*-values distinguishes no detectable enrichment from an underpowered test; we suggest this diagnostic should accompany any negative enrichment claim. We also identify the relationship that produces apparent convergence elsewhere: targeting multiplicity scales with 3′-UTR length (r = 0.63; ρ = 0.58), so “hub” genes are largely long-3′-UTR genes, an artefact well documented in mammalian miRNA target prediction [35,36,37] but rarely controlled in cross-kingdom work. Under permissive thresholds, this effect generated genes ostensibly regulated by almost the entire forage pool.

For the cross-kingdom field, this points to a practical analytical framework. Four elements—species-matched 3′-UTRs, per-kilobase 3′-UTR-length normalisation of scores, a background of targeted rather than all genes, and previously reported interactions as internal positive controls—are individually straightforward and each addresses a specific, documented confound. We did not run a formal ablation, so we do not claim that any single omission would by itself convert this result to a positive one; the point is that each element removes a known route by which permissive, human-surrogate analyses reach positive conclusions. The finding does not adjudicate the broader controversy [27,28,29,30,31,32,33,61]; it shows that a substantial part of the computational evidence adduced in its favour is explicable without invoking biology.

### 4.4. Candidates for Camel-Milk Biology and Applied Value

The candidate list translates the analysis into testable hypotheses. TCF7 and TCF7L2 sit at a Wnt–insulin node relevant to mammary metabolism, while the milk-associated candidates are headed by the prolactin and insulin receptors and the core lipogenic enzymes SCD, FASN, and THRSP [58], genes involved in setting milk-fat quantity and fatty-acid composition. That this set is not statistically enriched (*p* = 0.36) is stated plainly and constrains interpretation, but it does not remove the list’s practical utility: a small number of named genes with plausible mechanistic links is what a subsequent wet-laboratory or feeding study requires, and is more actionable than a diffuse genome-wide claim—provided the target transcripts are shown to be expressed in the relevant camel tissue, which the present analysis does not establish.

### 4.5. Does the Transfer Premise Hold? The State of the Evidence

A prediction of complementarity is only useful if dietary plant miRNAs can reach camel tissues at all, and the evidence here is predominantly negative. Controlled feeding studies in mice failed to detect meaningful intestinal uptake of plant miRNAs [14,31,32], and a primate experiment is more directly relevant: pigtail macaques fed a plant-rich meal containing abundant miRNAs showed no detectable miR160, miR166, miR167, miR168 or miR172 in blood at 1, 4 and 12 h, with droplet digital PCR yielding unreliable signals attributable to non-specific amplification [62]. Three of those families (miR166, miR167 and miR168) are among the sequences we find abundant in camel forage, so the same families that dominate the forage read set have been tested in a primate and did not appear in the circulation. Broader reviews reach the same conclusion, arguing that reported xenomiR signals largely reflect contamination, cross-mapping or sequencing artefacts and that absorbed copy numbers fall well below the level required to repress a transcript [33,61,63,64,65,66].

The case is not closed. It has been argued that extracellular vesicles may deliver cargo in ways that simple stoichiometric reasoning does not capture [67]. Our data cannot settle this dispute and were not designed to; what they do is remove one specific line of support for the positive case. If dietary plant miRNAs were shaping camel gene expression through coordinated targeting, a coordinated programme detectable by this species-matched, length-aware framework would be expected to leave a signal in the predicted target set. None was found. Combined with the uptake evidence, the reasonable working position is that direct, miRNA-mediated cross-kingdom regulation of the dromedary by forage miRNAs is unlikely to be a major physiological route, while remaining formally unresolved and leaving non-miRNA dietary effects untouched.

### 4.6. Implications for Camel Milk, Human Health, and Dryland Livestock

The applied stakes are real, because camel-milk extracellular vesicles are an active area of biomedical work. Vesicles purified from dromedary milk have been characterised in detail [68], and camel-milk exosomes have shown selective cytotoxicity toward hepatocellular and colorectal carcinoma cells while sparing normal cells [69,70] and have modulated oxidative stress and immunotoxicity in rats [71]; bovine milk vesicles, studied more extensively, survive gastrointestinal passage and alter gut microbiota and intestinal immunity [72,73,74], and whether camel vesicles behave identically is not yet established. Plant miRNAs from *M. oleifera* have separately been reported to affect immune, apoptotic, and lipid-metabolic pathways in mammalian cell systems [75,76,77,78], which is part of why the forage–milk question is asked at all. Camel milk is consequently being developed both as a functional food and as a vesicle source, which makes the determinants of its cargo scientifically important.

Our results speak to that question precisely because they are negative. If forage miRNAs travelled intact into milk and regulated consumer genes, the composition of camel-milk vesicles would depend on what the herd was browsing; we find no support for the underlying regulatory step. Independently, available characterisations suggest that dromedary milk miRNAs are largely of mammary origin rather than imported from the circulation [22,68], although direct tracing of their cellular source in the camel is still lacking; if borne out, this would mean that even successful intestinal uptake need not place plant sequences in milk. Two constraints would then act in series, and claims that browse composition alters camel milk through dietary miRNA transfer should be treated as unsupported until direct measurements exist.

This does not close the diet–milk question; it redirects it. Forage plainly influences milk, but the more plausible routes are nutritional and metabolic rather than nucleic-acid-mediated: fatty-acid precursors, phytochemicals, protein, and mineral supply, and their downstream effects on mammary gene expression. That route is almost uncharacterised in camels and is the one we would prioritise. The practical implications are correspondingly concrete. For dryland pastoral systems, forage-improvement programmes should be evaluated on milk composition and yield measured directly, not on inferred molecular mechanisms. For the emerging camel-milk vesicle work, cargo variation should be attributed to lactation stage, health status, and mammary physiology unless dietary transfer is demonstrated. And for the wider cross-kingdom field, the case shows how a species-matched analysis with internal positive controls can separate a real interaction from an artefact—a template applicable to any livestock species for which a genome and forage small-RNA data can be assembled.

### 4.7. Scope of the Present Inference

Several boundaries define what these results can and cannot establish, and most are addressed by a specific, feasible extension rather than being intrinsic to the approach. First, the analysis predicts sequence complementarity and does not test uptake: whether plant miRNAs survive foregut fermentation, cross the intestinal epithelium, reach the mammary gland, and occur at repressive stoichiometry remains open, and the quantitative objection to dietary miRNA function [31,32,34] applies here as elsewhere. The concentrated read set identified in Section 3.1 is what makes direct measurement tractable. Second, camel 3′-UTRs had to be reconstructed as CDS-to-transcript-end intervals because the current annotation lacks UTR features; this inherits transcript-end error, ignores alternative polyadenylation, and produced 362 implausibly long intervals that were excluded. Selecting the longest interval per gene enlarges the search space and is deliberately conservative for a negative result, but it is transcript-model dependent, and long-read mammary transcriptome sequencing would replace these estimates with experimentally defined 3′ ends—the single change that would most improve prediction accuracy. Third, requiring 5′-seed complementarity in addition to score and energy cut-offs, while appropriate for identifying strong sites, can miss genuinely plant-type non-seed sites and is therefore a source of false negatives; the non-recovery of miR159–TCF7 illustrates that false negatives occur at stringent settings. Fourth, functional annotation relies on human-derived gene-set libraries, so camel-specific biology such as water balance, thermotolerance, and lipid handling is under-represented; the negative result is accordingly stronger evidence against conserved mammalian programmes than against species-specific ones. Fifth, target predictions were made against a single female reference genome, and population-level 3′-UTR variation among dromedaries—which could create or abolish individual sites—was not assessed. Finally, the three forages are a tractable proxy rather than a survey of camel diets: they differ in cultivar, platform, and replication (one library for *Z. jujuba* against three each for the others), alfalfa is cultivated fodder rather than natural browse, and Sahelian browse species could not be included because no healthy-leaf small-RNA data exist for them. Generating such data for Acacia, Balanites, and Salvadora is an obvious next step, and the pipeline reported here applies to them unchanged.

### 4.8. Future Directions

The most informative next experiments are narrow rather than broad. Because five sequences carry half the pooled forage reads, targeted small-RNA sequencing and stem-loop qPCR for the five most abundant shared sequences (the miR159, miR166, miR396 and miR167 sequences), together with miR168 for its reported LDLRAP1 interaction, in plasma, mammary tissue, and milk extracellular vesicles of camels maintained on defined forage would test the transfer premise directly and at modest cost. Because plant-miRNA detection in mammalian matrices is exactly where the field has been misled, such assays should include synthetic spike-in standards, no-template and RNase controls, plant-specific sequence discrimination and sequencing confirmation, and detection in tissue should not by itself be read as dietary uptake. In parallel, quantifying expression of the candidate genes identified here (LDLRAP1, TCF7/TCF7L2, PRLR, INSR, SCD, FASN) in mammary tissue alongside milk fatty-acid profiling across controlled diets would establish whether forage composition correlates with milk phenotype at all, independently of any miRNA mechanism—an answer that matters to dryland dairy production whether or not cross-kingdom transfer occurs. Improved camel annotation and small-RNA data from true Sahelian browse would then allow the present analysis to be repeated under materially better conditions.

## 5. Conclusions

De novo, structure-based annotation defined compact 21-nt miRNA repertoires (170 loci; 116 mature sequences; 104 seed groups) for three arid-zone forage plants relevant to dromedary feeding, and revealed a strongly concentrated read set in which five sequences shared by three distantly related plant families carry half of all pooled forage reads, and miR159 alone contributes 31%. A stringent, length-aware, species-matched target analysis recovered the previously reported miR168a–LDLRAP1 pairing in the camel, demonstrating that the analysis can recover a known interaction, yet found no detectable enrichment of dietary plant miRNAs on the dromedary transcriptome at either threshold, with an approximately uniform *p*-value distribution giving no evidence of systematic enrichment under the tested framework. Apparent convergence under permissive settings was traced to 3′-UTR length, and the failure to recover the miR159–TCF7 pairing illustrates why an individual cross-kingdom interaction should not be assumed to transfer between mammalian species—a caution for the common practice of inferring livestock effects from human target predictions. The study delivers a reusable forage miRNA resource, a species-matched analytical framework for cross-kingdom inference, and a prioritised shortlist of five abundant sequences plus the candidate genes LDLRAP1, TCF7/TCF7L2, PRLR, and INSR, which together reduce the experimental investigation of desert forage and camel-milk biology to a tractable set of measurements.

## Figures and Tables

**Table 2:** Conserved miRNA families in the pooled forage read set, with the most abundant family (unassigned dominant sequence) shown for comparison. Abundance is the summed percentage of pooled reads across the seven libraries over all mature variants assigned to the family; “Variants” is the number of distinct mature sequences so assigned; “Species” lists the forage species in which the family was detected. Five exact mature sequences—the miR159 sequence, one miR166 variant, two miR396 variants, and one miR167 variant—are shared by all three species and together carry 51% of pooled reads. Sequences are given as DNA (T for U).

Family	Representative Mature Sequence (5′→3′)	Length	Abundance	Variants	Species
miR159	TTTGGATTGAAGGGAGCTCTA	21 nt	31.4%	1	all three
miR166	TCGGACCAGGCTTCATTCCCC	21 nt	10.9%	3	all three
miR396	TTCCACAGCTTTCTTGAACTT	21 nt	9.8%	2	all three
miR157	TTGACAGAAGATAGAGAGCAC	21 nt	5.8%	1	*M. oleifera*
miR167	TGAAGCTGCCAGCATGATCTG	21 nt	3.8%	3	all three
miR398	TGTGTTCTCAGGTCGCCCCTG	21 nt	1.4%	2	*M. sativa*, *Z. jujuba*
miR162	TCGATAAACCTCTGCATCCAG	21 nt	0.9%	1	*M. sativa*, *Z. jujuba*
unassigned	GGTGGACTGCTCGAGCTGCT	20 nt	15.5%	1	*M. oleifera*

**Table 3:** Internal positive controls: the two mammalian genes with previously reported regulation by a dietary plant miRNA, and their status in the camel-specific prediction. Recovery of one pairing shows that the analysis can detect a near-complementary site; non-recovery of the other is consistent with species-specific 3′-UTR divergence. “Camel gene targeted (any miRNA)” counts all plant miRNAs predicted to bind the gene and does not imply that the cognate pair was recovered.

Reported Mammalian Pair	Cognate miRNA in Forage	Camel Gene Targeted (Any miRNA)	Cognate Pair Recovered	Interpretation
miR168a → LDLRAP1 [17]	yes (exact match, *M. sativa*)	yes, 5 miRNAs	Yes	a near-complementary site is recovered at sequence level
miR159 → TCF7 [19]	yes (most abundant, all species)	yes, 14 miRNAs	No	consistent with 3′-UTR sequence divergence between species

**Table 4:** Curated milk-fat-synthesis and lactation genes among predicted camel targets (12 of 19). The set was not over-represented relative to the genome-wide targeting rate (Fisher’s exact odds ratio 1.68, *p* = 0.36); genes are listed as prioritised candidates for experimental testing, not as an enriched pathway. MFG, milk-fat globule.

Gene	Role in Lactation or Milk-Fat Synthesis	miRNAs	3′-UTR (nt)
*PRLR*	prolactin receptor; principal lactogenic signal	7	7085
*INSR*	insulin receptor; metabolic control of lipogenesis	7	4623
*SCD*	stearoyl-CoA desaturase; unsaturated milk fatty acids	6	3767
*FASN*	fatty-acid synthase; de novo fatty-acid synthesis	5	518
*THRSP*	Spot14; lipogenic transcriptional regulator	5	3947
*GPAM*	glycerol-3-phosphate acyltransferase; triacylglycerol synthesis	2	3655
*STAT5B*	prolactin signal transducer; lactogenesis	2	2444
*ACACA*	acetyl-CoA carboxylase α; rate-limiting lipogenic step	1	2114
*LPL*	lipoprotein lipase; fatty-acid uptake from circulation	1	1546
*XDH*	xanthine dehydrogenase; MFG secretion	1	554
*BTN1A1*	butyrophilin; MFG membrane	1	1193
*ACACB*	acetyl-CoA carboxylase β	1	189

## Data Availability

All primary data are publicly available. Small-RNA libraries: NCBI SRA SRR7770291–SRR7770293 (BioProject PRJNA488608), SRR4334534 (SRP090598) and SRR1201405–SRR1201407 (SRP040470). Reference assemblies: GCA_021397835.1 (*Moringa oleifera*), GCF_000826755.1 (*Ziziphus jujuba*), GCF_051527215.1 (*Medicago sativa*) and GCF_036321535.1 (*Camelus dromedarius*). All analysis code (the six numbered pipeline scripts for trimming, de novo annotation, 3′-UTR reconstruction, miRanda prediction, target aggregation and enrichment, plus the threshold-sweep and null-model scripts), all parameters, software and database versions, exact command lines, the fixed random seed (42) used for the null model, and intermediate result tables are deposited at https://github.com/maksimkamekspeks/camel-forage-mirna-crosskingdom (accessed on 24 August 2026), 10.5281/zenodo.21938711.

## Supplementary Materials

The following supporting information can be downloaded at: https://www.mdpi.com/article/10.3390/genes17091022/s1, Table S1: small-RNA datasets and read-processing quality control; Table S2: all 170 de novo miRNA loci with genomic coordinates, mature sequences, read support and Dicer calls; Table S3: all 9028 predicted camel target genes with Global Scores, length-normalised scores, targeting multiplicity, and 3′-UTR lengths; Table S4: curated milk-fat-synthesis and lactation genes among predicted targets; Table S5: complete GO Biological Process, KEGG, and MSigDB Hallmark over-representation results; Table S6: threshold-sensitivity sweep of targeting multiplicity and 3′-UTR-length correlation across miRanda score and energy cut-offs; Table S7: matched null-model results for multiplicity concentration and cross-species target overlap; Figure S1: robustness of the sparse-targeting and length-bias findings across thresholds and against the matched null.
